# Comparison of the Efficacy of 15- and 19-Week Chemotherapy Protocols Based on Vincristine, L-Asparaginase, Doxorubicin, and Prednisolone for Dogs with Multicentric Lymphoma

**DOI:** 10.3390/ani15040522

**Published:** 2025-02-12

**Authors:** Yi-Chen Lin, Shang-Lin Wang

**Affiliations:** 1 Graduate Institute of Veterinary Clinical Sciences, School of Veterinary Medicine, National Taiwan University, Taipei 10617, Taiwan; r11643006@ntu.edu.tw; 2 National Taiwan University Veterinary Hospital, College of Bioresources and Agriculture, National Taiwan University, Taipei 10672, Taiwan

**Keywords:** canine, chemotherapy, L-asparaginase, Lymphoma, outcome

## Abstract

Multicentric lymphoma is the most common type of hematopoietic neoplasm in dogs. Chemotherapeutic protocols typically consist of cyclophosphamide, doxorubicin, vincristine, and prednisolone (CHOP). The 15- and 19-week CHOP protocols exhibit similar outcomes, indicating that a shorter treatment duration may be a feasible treatment option. However, the 19-week LHOP protocol, which uses L-asparaginase instead of cyclophosphamide, results in a longer progression-free survival and a similar survival time compared to CHOP-based chemotherapy regimens. In this study, we conducted and historically compared the efficacy of the 15- and 19-week LHOP protocols to determine whether there are any differences in efficacy. Eighteen and twenty dogs underwent the 15- and 19-week LHOP protocols, respectively. No significant differences were found in age, body weight, sex, clinical stage, substage, T-cell phenotype, hypercalcemia status, and overall response rate between the two groups. The time to progression (TTP) and lymphoma-specific survival (LSS) for the 19-week LHOP protocol were significantly longer than those for the 15-week LHOP protocol (*p* = 0.004 and *p* = 0.008, respectively). Thus, the 19-week LHOP protocol may be a better treatment option for dogs with multicentric lymphoma. However, the 15-week LHOP protocol lowered the treatment cost and reduced the treatment time.

## Simple Summary

We conducted and historically compared the efficacy of the 15- and 19-week LHOP protocols (L-asparaginase, doxorubicin, vincristine, and prednisolone) to determine whether there are any differences in efficacy. Eighteen and twenty dogs underwent the 15- and 19-week LHOP protocols, respectively. No significant differences were found in age, body weight, sex, clinical stage, substage, T-cell phenotype, hypercalcemia status, and overall response rate between the two groups. The time to progression (TTP) and lymphoma-specific survival (LSS) for the 19-week LHOP protocol were significantly longer than those for the 15-week LHOP protocol (*p* = 0.004 and *p* = 0.008, respectively). Thus, the 19-week LHOP protocol may be a better treatment option for dogs with multicentric lymphoma. However, the 15-week LHOP protocol lowered the treatment cost and reduced the treatment time.

## 1. Introduction

Lymphoma is the most common hematopoietic neoplasm that develops in dogs, accounting for 7–24% and 83% of all canine tumors and hematopoietic malignancies, respectively [1,2]. The most common type is multicentric lymphoma, which is characterized by generalized lymphadenopathy [3]. Studies investigating chemotherapy protocols for the treatment of canine lymphoma aimed to improve progression-free survival, survival time, and quality of life. CHOP-based chemotherapies (C, cyclophosphamide; H, doxorubicin; O, vincristine; and P, prednisolone) are the most commonly used protocols for high-grade lymphoma. Such protocols have achieved remission rates of >85% and survival times ranging from 7 to 12 months [4,5,6,7].

The median duration of the first remission and survival time for the 19-week CHOP protocol was 174 days and 275 days, respectively [8]. A recent study investigating the 15-week CHOP protocol achieved a median progression-free interval of 140 days and a median overall survival time of 257 days [4]. Another study published in 2024 showed that the median progression-free survival and median overall survival time were 196 days and 302 days, respectively, in the 19-week CHOP protocol. The median progression-free survival and median overall survival time were 209 days and 321 days, respectively, in the 25-week CHOP protocol. There was no significant difference in the progression-free survival and overall survival time between the two protocols [9]. The various treatment durations of CHOP-based protocols from the traditional 25 weeks to the current 19 or 15 weeks all achieved similar outcomes [8,9,10]. However, a shorter treatment duration could lower costs and treatment time and achieve comparably satisfactory results, making it more feasible and acceptable for dog owners.

One study evaluated a 19-week LHOP protocol that used L-asparaginase instead of the traditional cyclophosphamide and resulted in a longer progression-free survival but similar overall survival time to those of CHOP-based chemotherapies [11]. Furthermore, the adverse effects of L-asparaginase reported in the study were well tolerated and self-limiting. Therefore, the 19-week LHOP chemotherapy protocol was established as another chemotherapeutic option for canine large-cell multicentric lymphoma.

Canine lymphoma is not curable; thus, owners may hesitate to commit the time and finances to complete a relatively lengthy multi-drug protocol [10,12]. This study compared the response rate, time to progression (TTP), and lymphoma-specific survival (LSS) between the 15- and 19-week LHOP regimens for dogs with large-cell multicentric lymphoma. We hypothesized that the 15- and 19-week LHOP chemotherapy protocols can achieve comparable results in canine multicentric lymphoma.

## 2. Materials and Methods

### 2.1. Patient Selection and Evaluation

#### 2.1.1. Fifteen-Week LHOP Protocol

This study enrolled dogs diagnosed with large-cell multicentric lymphoma either by cytology or histopathology at the National Taiwan University Veterinary Hospital from August 2021 to March 2024. The inclusion criteria were dogs with multicentric lymphoma who underwent the 15-week LHOP chemotherapy protocol (Table 1). We collected data on breed, sex, neutered status, age, body weight, body temperature, disease stage and substage, immunophenotype, complete blood cell count, biochemistry panel, treatment response, start date of therapy, date of relapse, and date of death. The immunophenotype was determined by flow cytometry or immunohistochemistry. Canine lymphoma was staged according to the World Health Organization (WHO) staging system based on clinical presentation, radiography, ultrasonography, and bone marrow aspirate cytology to evaluate the anatomical site involved and the clinical signs expressed [13]. Dogs that had previously received chemotherapy or corticosteroid treatment were excluded. This study was approved by the Institutional Animal Care and Use Committee of the National Taiwan University (Approval number: NTU110-EL-00117). All the owners had read and signed the consent form before participating in the study.

#### 2.1.2. Nineteen-Week LHOP Protocol

Data on dogs with large-cell multicentric lymphoma that underwent the 19-week LHOP chemotherapy protocol at the National Taiwan University Veterinary Hospital between September 2018 and October 2020 were retrospectively reviewed and analyzed (Table 1). Dogs that had received chemotherapy or corticosteroid treatment prior to starting the 19-week LHOP chemotherapy protocol were excluded. All dogs were involved in the previous research and continued follow-ups until March 2024 or until the patient’s death [11].

### 2.2. Treatment Response Assessment and Follow-Up Evaluation

Treatment response was determined using the Veterinary Cooperative Oncology Group consensus criteria for assessing peripheral lymph nodes when the dogs returned for every treatment [14]. Complete response (CR) was defined as the complete resolution of all measurable diseases. Partial response (PR) was defined as a decrease of >30% but <100% in the mean sum of the longest diameter of the target lesions. Stable disease (SD) was defined as a decrease of <30% or an increase of <20%, while progressive disease was defined as an increase of >20% or the development of a new lesion.

TTP was determined from the date of therapy initiation to the date of progression. LSS was calculated from the initiation of chemotherapy to the date of patient death from lymphoma. Dogs were censored from the TTP analysis if they did not relapse before the end of the study period, were lost to follow-up during remission, or died before relapse. Dogs were censored from the LSS analysis if they were alive, lost to follow-up, or died from causes other than lymphoma at the end of the study.

The follow-up evaluation interval for both treatment groups after the completion of induction chemotherapy was once monthly in the first 3 months and then every 3 months thereafter. Furthermore, the owners were informed that they could visit the clinic anytime if disease progression was observed.

### 2.3. Rescue Protocols

The chemotherapeutic protocol implemented in case of tumor relapse in both groups was as follows: The dogs who completed the preceding induction chemotherapy received the same protocol upon relapse (reinduction), except that the doxorubicin was replaced with mitoxantrone (5 mg/m^2^). Otherwise, the dogs received single-agent lomustine with prednisolone as rescue therapy for tumor relapse during induction or reinduction chemotherapy.

### 2.4. Statistical Analysis

The Shapiro–Wilk test was used to check the normality of the distribution. Student’s t-test or the Mann–Whitney test was used to compare age and body weight between the 15- and 19-week LHOP protocols. Fisher’s exact test was used to analyze the differences in sex, clinical stage and substage, immunophenotype, hypercalcemia, and response rate between the two protocols. The median TTP and LSS were determined by Kaplan–Meier analysis, and the differences between the two groups were assessed using the log-rank test. A *p*-value < 0.05 was considered statistically significant. Statistical calculations were performed using a commercial statistical software package (IBM SPSS Statistics version 29).

## 3. Results

### 3.1. Fifteen-Week LHOP Protocol

Eighteen dogs were included in the 15-week LHOP group, which consisted of five Welsh Corgis, three mixed breeds, three Poodles, two Dachshunds, two French Bulldogs, and one each of Beagle, Pomeranian, and Shih Tzu. The median age was 9.5 years (range: 3–15 years), and the median body weight was 10.9 kg (range: 4.2–18.4 kg). Twelve dogs were male, seven of which were castrated, while six were female, four of which were spayed. Three dogs were diagnosed with WHO stage V, twelve with stage IV, two with stage III, and one with stage II. Eight dogs exhibiting clinical signs were classified as substage b, while the remaining ten dogs were classified as substage a. Seventeen dogs were diagnosed with B-cell lymphoma, while one dog was diagnosed with T-cell lymphoma. One dog was diagnosed with hypercalcemia concurrent with T-cell lymphoma before undergoing chemotherapy, after which its calcium levels normalized.

The response rate of the 15-week LHOP group was 100%, with seventeen dogs exhibiting CR and one exhibiting PR. Sixteen dogs exhibited CR after receiving vincristine on week 1, and one dog exhibited CR after receiving L-asparaginase on week 2. The average treatment was 1.06 times to achieve CR. Two dogs died (one from renal disease and one from heart disease) without evidence of tumor progression and were subsequently censored from the TTP analysis. Seventeen dogs completed the induction chemotherapy, while one dog relapsed before completion. Fifteen dogs and one dog received reinduction chemotherapy and rescue therapy, respectively, for the first relapse. Four dogs were censored from the LSS analysis. Two dogs died from other diseases (one from renal disease and one from heart disease), while two others survived until the end of the study. The other fourteen dogs died of lymphoma. The median TTP and LSS were 265 days (range: 91–437 days) and 420 days (range: 91–663 days), respectively.

### 3.2. Nineteen-Week LHOP Protocol

Twenty dogs were included in the 19-week LHOP group, including seven mixed breeds, three Maltese Terriers, two Chihuahuas, and one each of Beagle, Welsh Corgi, Dachshund, French Bulldog, Golden Retriever, Standard Poodle, Pug, and Yorkshire Terrier. The median age was 9.5 years (range: 4–14 years). The median body weight was 9.55 kg (range: 2.2–37.7 kg). Eight dogs were male, three of which were castrated, while twelve were female, ten of which were spayed. Two dogs were diagnosed with WHO stage V, fifteen with stage IV, and three with stage III. Seven dogs exhibiting clinical signs were classified as substage b, while the remaining thirteen dogs were classified as substage a. Seventeen dogs were diagnosed with B-cell lymphoma, while three dogs were diagnosed with T-cell lymphoma. No dogs had hypercalcemia before undergoing chemotherapy.

The response rate of the 19-week LHOP group was 100%, with eighteen dogs exhibiting CR and two exhibiting PR. Three dogs did not relapse, one was lost to follow-up, and five died without evidence of tumor progression. All these cases were censored from the TTP analysis. Eighteen dogs completed the induction chemotherapy, one relapsed, and one was lost during induction chemotherapy. Ten dogs and one dog received reinduction chemotherapy and rescue therapy, respectively, for the first tumor relapse. Nine dogs were censored from the LSS analysis. Six dogs died from other diseases (four from heart disease and two from renal disease), one dog was lost to follow-up, and two dogs survived until the end of the study. The other eleven dogs died of lymphoma. The median TTP and median LSS were 401 days (range: 28–1435 days) and 530 days (range: 71–1435 days), respectively.

### 3.3. Comparison Between the 15-Week and 19-Week LHOP Protocols

**Figure 1:**
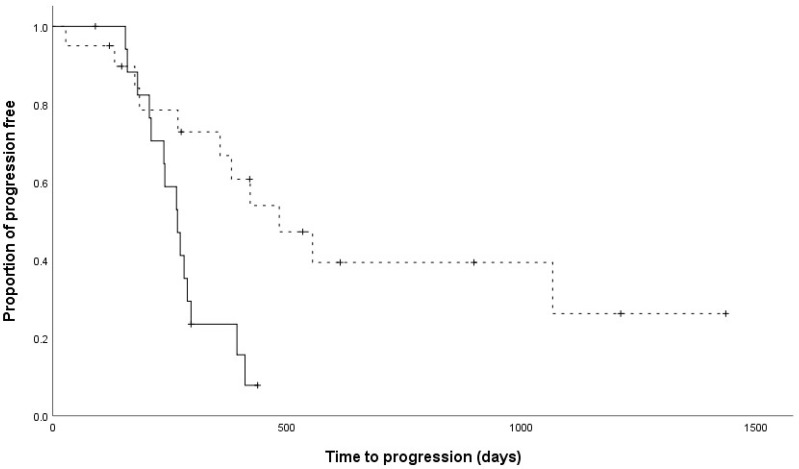
Kaplan–Meier curves of the time to progression for dogs with lymphoma. Dogs that received the 15-week LHOP chemotherapy are indicated by a solid line, whereas dogs that received the 19-week LHOP chemotherapy are indicated by a dashed line (*p* = 0.004). Tick marks indicate censored patients. The median times to progression were 265 days and 401 days in the 15-week and 19-week LHOP protocols, respectively.

**Figure 2:**
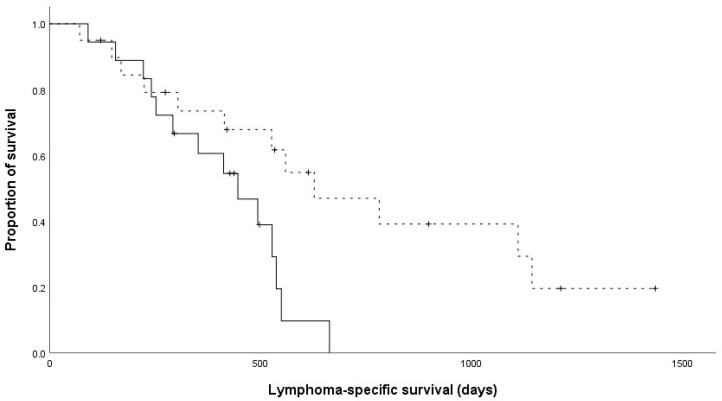
Kaplan–Meier curves of the lymphoma-specific survival for dogs with lymphoma. Dogs that received the 15-week LHOP chemotherapy are indicated by a solid line, whereas dogs that received the 19-week LHOP chemotherapy are indicated by a dashed line (*p* = 0.008). Tick marks indicate censored patients. The median lymphoma-specific survival were 420 days and 530 days in the 15-week and 19-week LHOP protocols, respectively.

No significant differences were found in age (*p* = 0.851), body weight (*p* = 0.934), sex (*p* = 0.093), clinical stage (*p* = 0.448), substage (*p* = 0.396), T-cell phenotype (*p* = 0.344), hypercalcemia status (*p* = 0.474), or overall response rate (*p* = 1) between the two groups (Table 2). However, the TTP and LSS for the 19-week LHOP protocol were significantly longer than those for the 15-week LHOP protocol (*p* = 0.004 and *p* = 0.008, respectively) (Figure 1 and Figure 2).

## 4. Discussion

Cyclophosphamide is a foundational chemotherapy drug that is used in combination regimens for treating lymphoma in both humans and small animals. It is an alkylating agent that alkylates or binds to DNA, causing the cross-linking of DNA and RNA strands and inhibiting protein synthesis [15]. A study evaluated the efficacy of each cytotoxic drug in the CHOP regimen using real-time polymerase chain reaction to quantify the residual tumor cells after each treatment. The analysis revealed that cyclophosphamide had a lower cytoreductive effect than vincristine and doxorubicin [16]. Furthermore, relapse in canine lymphoma frequently occurs after cyclophosphamide administration [17]. Therefore, substituting cyclophosphamide with other drugs may be more therapeutically effective in canine lymphoma.

Vincristine works at the M phase of the cell cycle to disrupt the mitotic spindle apparatus and causes metaphase arrest and cytotoxicity [18]. Cyclophosphamide and doxorubicin mainly function at the S phase and may have overlapping cytotoxic effects on tumor cells [19,20]. L-asparaginase is an enzyme derived from bacteria that inhibits the uptake of L-asparagine into lymphoid tumor cells, thereby impairing protein synthesis and inducing cell death [21]. L-asparaginase is mostly active at the G1 phase and may have a synergic effect with doxorubicin and vincristine, thereby inducing more lymphoma cell deaths and conferring longer remission times [11,22]. Despite its efficacy, L-asparaginase can cause allergic reactions and pancreatitis in both humans and dogs. Pretreatment with steroids or diphenhydramine can reduce hypersensitivity reactions [23,24]. However, recent studies have found no significant increase in the incidence of canine pancreatic lipase immunoreactivity before and after L-asparaginase treatment [25]. In addition, the LHOP protocol may also be less myelosuppressive because of the replacement of the cyclophosphamide by the L-asparaginase, which is not myelosuppressive. The substitution of L-asparaginase into the 19-week CHOP protocol did not worsen bone marrow suppression or prolong the treatment duration. Therefore, L-asparaginase is a relatively safe drug with fewer side effects and a good chemotherapeutic agent candidate for the treatment of canine lymphoma. The 19-week LHOP chemotherapy protocol provided a better progression-free survival than and a comparable survival time to the 19-week CHOP protocol [11].

Hawkes et al. reported a study that compared the outcomes of dogs with lymphoma treated with 19-week and 25-week CHOP protocols. Five hundred and two dogs from 16 European oncology referral centers were included in the study. No significant difference in progression-free survival and overall survival time was found between the two protocols. Therefore, a short protocol may be a reasonable standard of care for future clinical trials [9]. In Curran and Thamm’s study, 134 dogs with naïve canine multicentric lymphoma underwent a 15-week CHOP protocol. It achieved an overall response rate of 98%, a median progression-free survival time of 176 days, and a median survival time of 311 days [10]. Previous studies have demonstrated that reducing the number of vincristine doses by four in the 19-week CHOP protocol provides a similar treatment outcome, thereby establishing the 15-week CHOP protocol as a feasible treatment option for canine lymphoma [26,27]. Furthermore, this prompted our comparison between the 19- and 15-week LHOP protocols in the present study.

Reducing the treatment duration from 19 to 15 weeks was more convenient for the pet owners. The 15-week LHOP protocol lowered the overall treatment cost because it required fewer weeks of therapy and the performance of fewer blood tests before treatment. Reducing the treatment duration from 19 to 15 weeks at our hospital decreased the total cost by approximately 20%, which represents significant financial relief for pet owners.

Previous studies have demonstrated that the 19-week CHOP protocol caused gastrointestinal adverse effects with grades 1 and 2 toxicity in 80% of dogs and with grade 4 toxicity in 7% of dogs [8]. Although the 15-week CHOP study focused on the total number of gastrointestinal adverse effects, no cases of grade 4 gastrointestinal toxicity were recorded [10]. The incidence of grade 4 neutropenia was 10% and 7% for the 19- and 15-week CHOP protocols, respectively, in the studies mentioned previously. Although treatment toxicity was not evaluated in our study, the four-dose reduction in vincristine treatment in the 15-week LHOP protocol may potentially reduce the adverse effects and toxicity associated with bone marrow suppression and gastrointestinal issues. Further larger prospective studies are needed to verify this hypothesis.

However, reducing the treatment duration, relative intensity, and administration frequency of the cytotoxic drug may have affected the therapeutic efficacy and caused the significant differences in TTP and LSS between the two treatment groups. Vincristine exhibits strong cytoreductive activity in canine lymphoma by breaking down cytoplasmic microtubules, inducing cell cycle arrest at the G2 to M phase transition [16]. In the present study, reducing vincristine treatment by four doses may have lowered the TTP and LSS in the 15-week LHOP protocol. Our results were not consistent with those of Curran and Thamm’s investigation of the efficacy of a 15-week CHOP protocol in canine lymphoma. In their study, reducing vincristine treatments by four doses from the 19-week to the 15-week CHOP protocol resulted in 176 days of progression-free survival and 311 days of overall survival time [10]. The progression-free survival and overall survival time of the 15-week CHOP protocol were similar to the 174 days of progression-free survival and 275 days of overall survival time in the 19-week CHOP protocol reported by Hosoya et al. (2007). A possible explanation may be the differences in the cytotoxic drugs used in the protocols. Cyclophosphamide is an alkylating agent that acts by cross-linking DNA strands and interferes with DNA replication and protein synthesis [19]. L-asparaginase is an enzymatic antineoplastic agent that acts by depleting extracellular asparagine, resulting in inhibited protein synthesis and eventual cell apoptosis [27]. The synergistic effects of prednisolone, vincristine, and doxorubicin may differ when combined with cyclophosphamide or L-asparaginase. Further studies are needed to verify this possibility. Nevertheless, the 19-week LHOP protocol achieved significantly longer TTP and LSS than the 15-week LHOP protocol. Thus, the 19-week LHOP chemotherapy protocol achieved better outcomes in treating canine multicentric lymphoma.

Many prognostic factors have been reported in dogs with lymphoma, such as tumor stage and substage, immunophenotype, hypercalcemia, anemia, thrombocytopenia, histopathology grade, mitotic index, anatomic location, proliferating cell nuclear antigen, aneuploidy, and proliferation indices [28,29,30]. We only collected and compared the data on disease stage and substage, immunophenotype, and hypercalcemia between the two treatment groups, which showed no significant differences. However, most dogs were diagnosed by lymph node cytology without histopathology or further examination. Therefore, some of the above prognostic factors were not evaluated and compared between the two treatment groups, which may have influenced the outcomes.

Patients’ concurrent diseases must be considered when choosing chemotherapy protocol. Vincristine can cause ileus and doxorubicin can cause cumulative cardiotoxicity [31,32]. L-asparaginase may cause anaphylactic reactions or pancreatitis in some patients; therefore, pre-treatment prevention and post-treatment monitoring should be conducted [33]. Metronomic chemotherapy is a low-dose, long-term, and frequently administered chemotherapy that shows clinical benefits for several kinds of cancer patients without severe toxicity [34]. Oral administration, few side effects, and low cost are key benefits of this therapy. However, to the best of our knowledge, there have been no studies that used metronomic chemotherapy to treat canine large-cell lymphoma. Multi-drug combination chemotherapy is still the main treatment for canine multicentric lymphoma.

This study has some limitations. The number of dogs with lymphoma in both of the treatment groups was small; future larger randomized studies are required to confirm the results reported. While the rescue protocol was standardized, it was modified according to the patient’s response and the owner’s preference thereafter, which may have caused the differences in LSS between the two groups. The side effects of chemotherapy were not recorded; this information may provide valuable information for owners in choosing a chemotherapeutic protocol.

## 5. Conclusions

The 15-week LHOP protocol lowered the treatment cost and reduced treatment duration. However, it also significantly decreased the TTP and LSS compared with the 19-week LHOP protocol. Based on the outcomes, the 19-week LHOP protocol may be a better option for the treatment of dogs with multicentric lymphoma.

## Figures and Tables

**Table 1:** 15-week and 19-week LHOP chemotherapy protocol.

	**Week**																		
15-week LHOP	1	2	3	4	5	6	7	8	9	10	11	12	13	14	15	16	17	18	19
Vincristine	**●**				**●**				**●**				**●**						
(0.7 mg/m^2^ IV)																			
L-asparaginase		**●**				**●**				**●**				**●**					
(400 U/kg IM)																			
Doxorubicin			**●**				**●**				**●**				**●**				
(30 mg/m^2^ IV or 1 mg/kg IV)																			
Prednisolone	2	1.5	1	0.5															
(mg/kg/day PO)																			
	**Week**																		
19-week LHOP	1	2	3	4	5	6	7	8	9	10	11	12	13	14	15	16	17	18	19
Vincristine	**●**		**●**			**●**		**●**			**●**		**●**			**●**		**●**	
(0.7 mg/m^2^ IV)																			
L-asparaginase		**●**					**●**					**●**					**●**		
(400 U/kg IM)																			
Doxorubicin				**●**					**●**					**●**					**●**
(30 mg/m^2^ IV or 1 mg/kg IV)																			
Prednisolone	2	1.5	1	0.5															
(mg/kg/day PO)																			

**Table 2:** Comparisons on signalment, negative prognostic factors, response, time to progression, and lymphoma-specific survival between the 15-week LHOP and the 19-week LHOP treatment groups.

	15-Week LHOP	19-Week LHOP	*p*-Value
	(n = 18)	(n = 20)	
Mean age (years)	9.3	9.5	0.851
Mean body weight (kg)	11.1	11.3	0.515
Sex			0.093
Female	6 (33.3%)	12 (60%)	
Male	12 (66.7%)	8 (40%)	
Clinical stage V	3 (16.7%)	2 (10%)	0.448
Substage b	8 (44.4%)	7 (35%)	0.396
Tcell	1 (5.6%, 1/18)	3 (15%, 3/20)	0.344
Hypercalcemia	1 (5.6%, 1/18)	0 (0%)	0.474
Overall response (CR + PR)	18 (100%)	20 (100%)	1
Median TTP (days)	265 (91–437 days)	401 (28–1435 days)	0.004
Median LSS (days)	420 (91–663 days)	530 (71–1435 days)	0.008

CR, complete remission; PR, partial remission; TTP, time to progression; LSS, lymphoma-specific survival.

## Data Availability

The original contributions presented in the study are included in the article, further inquiries can be directed to the corresponding author.
